# Growth, productivity, and relative extinction risk of a data-sparse devil ray

**DOI:** 10.1038/srep33745

**Published:** 2016-09-23

**Authors:** Sebastián A. Pardo, Holly K. Kindsvater, Elizabeth Cuevas-Zimbrón, Oscar Sosa-Nishizaki, Juan Carlos Pérez-Jiménez, Nicholas K. Dulvy

**Affiliations:** 1 Earth to Ocean Research Group, Department of Biological Sciences, Simon Fraser University, Burnaby, BC, Canada; 2 Department of Ecology, Evolution, and Natural Resources, Rutgers University, New Brunswick, NJ, USA; 3 Fisheries Biology Consultant, Boulevard Chahue #66 Mz 6, Sector R, La Crucecita, Bahías de Huatulco, Oaxaca, Mexico; 4 Laboratorio de Ecología Pesquera, Departamento de Oceanografía Biológica, CICESE, Ensenada, Baja California, Mexico; 5 Ciencias de la Sustentabilidad, El Colegio de la Frontera Sur, Lerma, Campeche, Mexico

## Abstract

Devil rays (*Mobula* spp.) face intensifying fishing pressure to meet the ongoing international demand for gill plates. The paucity of information on growth, mortality, and fishing effort for devil rays make quantifying population growth rates and extinction risk challenging. Furthermore, unlike manta rays (*Manta* spp.), devil rays have not been listed on CITES. Here, we use a published size-at-age dataset for the Spinetail Devil Ray (*Mobula japanica*), to estimate somatic growth rates, age at maturity, maximum age, and natural and fishing mortality. We then estimate a plausible distribution of the maximum intrinsic population growth rate (*r*_*max*_) and compare it to 95 other chondrichthyans. We find evidence that larger devil ray species have low somatic growth rate, low annual reproductive output, and low maximum population growth rates, suggesting they have low productivity. Fishing rates of a small-scale artisanal Mexican fishery were comparable to our estimate of *r*_*max*_, and therefore probably unsustainable. Devil ray *r*_*max*_ is very similar to that of manta rays, indicating devil rays can potentially be driven to local extinction at low levels of fishing mortality and that a similar degree of protection for both groups is warranted.

Understanding the sustainability and extinction risk of data-sparse species is a pressing problem for policy-makers and managers. This challenge can be compounded by economic, social and environmental actions, as in the case of the mobulid rays (family Mobulidae). This group includes two species of charismatic and relatively well-studied manta rays (*Manta* spp.), which support a circumtropical dive tourism industry with an estimated worth of $73 million USD per year^1^. The Mobulidae also includes nine described species of devil rays (*Mobula* spp.). The recent international trade regulation of manta ray gill plates under the Convention on International Trade in Endangered Species of Wild Fauna and Flora (CITES)^2^ may shift gill plate demand from manta rays onto devil rays.

Devil rays are increasingly threatened by target and incidental capture in a wide range of fisheries, from small-scale artisanal to industrial trawl and purse seine fisheries targeting pelagic fishes^3^,^4^. The meat is sold or consumed domestically, and the gill plates are exported, mostly to China, to be consumed as a health tonic^5^. Small-scale subsistence and artisanal fisheries, mainly for meat, have operated throughout the world for decades^5^. For example, devil rays were caught by artisanal fishermen using harpoons and gill nets around Bahia de La Ventana, Baja California, Mexico, until 2007 when the Mexican government prohibited the take of mobulid rays^6^.

Overall, around 90,000 devil rays are estimated to be caught annually in fisheries worldwide^7^. Many industrial fleets capture devil rays incidentally. For example, European pelagic trawlers in the Atlantic catch a range of megafauna including large devil rays at a rate of up to one individual per hour^3^, while purse seine fleets targeting tunas capture tens of thousands of devil rays each year^4^. Even if devil rays are handled carefully and released, their post-release mortality might be significant^8^. We do not know whether current fishing pressure and international trade demand for devil rays are significant enough to cause population declines and increase extinction risk. The degree to which devil ray populations can withstand current patterns and levels of fishing mortality depends on their intrinsic productivity, which determines their capacity to replace individuals removed by fishing.

Slow somatic growth and large body size are associated with low productivity and elevated threat status and extinction risk in marine fishes, including elasmobranchs^9^,^10^,^11^. Based on these correlations, the American Fisheries Society developed criteria to define productivity and extinction risk. They defined four levels of productivity (very low, low, medium, and high) based on four life history traits (age at maturity, longevity, fecundity, and growth rate, which is related to the von Bertalanffy growth coefficient *k*) and the intrinsic rate of population increase *r*^9^. According to these criteria, manta rays have very low or low productivity, with some of the lowest maximum rates of population increase (*r*_*max*_) of any shallow-water chondrichthyan^9^,^12^.

Here we evaluate the productivity, and hence relative extinction risk of large devil rays, using the only age and growth study available for this group^13^. We use a Bayesian approach to estimate somatic growth rate and a demographic model based on the Euler-Lotka equation to calculate the maximum intrinsic rate of population increase (*r*_*max*_) for a population of the Spinetail Devil Ray *Mobula japanica* (Müller & Henle, 1841), which we compare to the productivity of 95 other sharks, rays, and chimaeras.

## Methods

We use the only study to measure length-at-age for catch data of *M. japanica*^13^. The Spinetail Devil Ray is similar in life history and size to other exploited mobulids, so we assume it is representative of the relative risk of the group. Spinetail Devil Rays examined in this study were caught seasonally by artisanal fishers using harpoon and gill nets around Punta Arenas de la Ventana, Baja California Sur, Mexico, during the summers of 2002, 2004, and 2005^13^.

First, we estimate growth parameters using a Bayesian approach that incorporates prior knowledge of maximum size and size at birth of this species, using the length-at-age data presented in Cuevas-Zimbrón *et al.*^13^ (Part 1). Second, we use the same dataset to plot a catch curve of the relative frequency of individuals in each age-class, from which we can infer a total mortality rate (*Z*) that includes both fishing (*F*) and natural mortality (*M*) (Part 2). This places an upper bound on our estimate of natural mortality, and allows us to compare the observed rate of mortality for this population with independent estimates of natural mortality rates. Third, we estimate the maximum intrinsic rate of population increase (*r*_*max*_) for this devil ray (Part 3) and compare it against the *r*_*max*_ of 95 other chondrichthyans, calculated using the same method (Part 4).

### Part 1: Re-estimating von Bertalanffy growth parameters for the Spinetail Devil Ray

We analyse a unique set of length-at-age data for a single population of *M. japanica* caught in a Mexican artisanal fishery. Individuals in this sample were limited to 1100 and 2400 mm disc width (DW), which falls short (77%) of the maximum disc width reported elsewhere^14^. Therefore, we use a Bayesian approach to refit growth curves to this length-at-age dataset^15^, using published estimates of maximum size and size at birth to set informative priors. Our aim is to reconstruct the growth rate of the species, rather than this local population. Hence, we focus on finding the growth rate estimate that would be most consistent with the species maximum size and the available size-at-age data.

We fit the three-parameter von Bertalanffy equation to the length-at-age data, combining sexes:


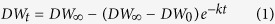


where *DW*_*t*_ is disc width at age *t*, and the growth coefficient *k*, disc width-at-age zero *DW*_0_, and asymptotic width *DW*_∞_ are the von Bertalanffy growth parameters. These parameters are conventionally presented in terms of length; however disc width is the appropriate measurement for these rays and we explicitly note all our size estimates as disc width to avoid any confusion.

In order to account for multiplicative error, we log-transformed the von Bertalanffy growth equation and added an error term:


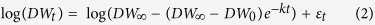


This can be written as:


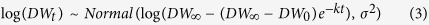


A Bayesian approach allows us to incorporate expert knowledge using prior distributions of estimated parameters. We based our informative priors on our knowledge of maximum disc widths and size-at-birth of *M. japanica*. Reported size at birth ranges from 880 to 930 mm DW^16^,^17^, while reported maximum size for *M. japanica* is 3100 mm DW^18^. While this reported maximum size is from an individual caught in New Zealand, we use this estimate as genetic evidence suggests that the Spinetail Devil Rays has little genetic substructure throughout the Pacific Ocean^19^. Nonetheless, growth and size differences could arise from environmental factors (e.g., temperature differences), which could lead to biased growth coefficient estimates in our Bayesian model.

Asymptotic size can be estimated from maximum size in fishes using the following equation^20^:


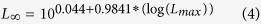


where *L*_*max*_ is maximum size, in centimetres. The data used to estimate this relationship included some species whose size was estimated in disc width, thus we use for this devil ray. This results in an estimate of *DW*_∞_ = 1.01 * *DW*_*max*_ for a value of *DW*_*max*_ = 3100 mm. Instead of setting a fixed value for the conversion parameter, we create a hyperprior for this parameter, defined as *kappa*, based on a gamma distribution around a mean of 1.01. We concentrated the probability distribution of *kappa* between 0.9 and 1.1, and fully constrain it between 0.7 and 1.3^20^:


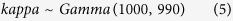


We also constrain our prior for *DW*_0_ around size at birth, and use a beta distribution to constrain our prior for growth coefficient *k* between zero and one, with a probability distribution that is slightly higher closer to a value of 0.1:


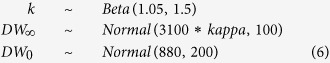


We compared the effect of our informative priors on our posteriors with parameter estimates with weaker priors, in which we maintained the mean of the distributions but increased their variance:


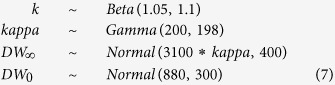


We also considered a scenario with uninformative priors, where all prior distributions are uniform:


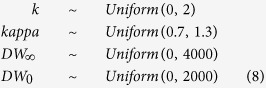


In all models we set an weakly informative prior for the variance *σ*^2^, such that:


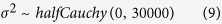


A summary of the priors used can be seen in Table 1. Bayesian inference was conducted using RStan v2.7.0^21^,^22^ running in R v3.2.1^23^.

### Part 2. Estimating total mortality using the catch curve

The length-at-age dataset of *M. japanica* can be used as a representative sample of the number of individuals within each age-class if we assume that sampling was opportunistic, and non-selective across each age- or size-class. We also assume that there is limited migration in and out of this population. With these assumptions, counting the number of individuals captured in each age-class represents the population age structure, which can be used to construct a catch curve. We further consider the validity of these assumptions in the Results and Discussion.

Catch curves are especially useful for data-poor species lacking stock assessments^24^,^25^. The frequency of individuals in older or larger classes decreases due to a combination of natural and fishing mortality. If fishing is non-selective with respect to size, the total mortality rate *Z*, which is a combination of both fishing mortality *F* and natural mortality *M*, can be estimated using a linear regression as the slope of the natural log of the number of individuals in each class^26^. This information is very valuable when inferring whether fishing mortality *F* is unsustainable. We calculate *Z* as the slope of the regression of the catch curve, including only those ages or sizes that are vulnerable to the fishery. There were no devil rays aged 12 or 13, and therefore these age-classes were removed from the catch curve analysis before fitting each regression. Because these age classes are some of the oldest, removing these points is likely to prevent the overestimation of total mortality.

We removed age-classes that had zero individuals in our sample to be able to take the natural logarithm of the count. Because there is uncertainty associated with the dataset (due to its relatively small size), we resampled a subset of the dataset 20,000 times, after randomly removing 20% of the points. This allowed us to quantify uncertainty in our estimate of *Z*. For each subset, we computed the age-class with the maximum number of samples, and removed all age-classes younger than this peak. With the remaining age-classes, we fit a linear regression to estimate the slope which is equivalent to −*Z*. This method for estimating mortality relies on two assumptions of the selectivity of the fishery. First, catch is not size-selective once individuals are vulnerable to the fishery. Second, if young age-classes are less abundant than older age-classes, they are assumed to have lower catchability. This is why we removed the younger age-classes before the “peak” abundance of each sample, as this will affect the steepness of the slope.

Given that the *M. japanica* length-at-age dataset has very few individuals aged 10 and older, we repeated the bootstrapping approach excluding ages 10 and older in order to avoid overestimating *Z*. This means that our range of Z estimates incorporate the possibility that the oldest individuals are missing from our sample because of migration, rather than fishing mortality, with the resulting estimate being a more conservative estimate of total mortality.

### Part 3. Estimating *M. japanica* maximum population growth rate

Maximum intrinsic population growth rates *r*_*max*_ can be estimated based on a simplified version of the Euler-Lotka equation^27^,^28^. We use the an updated method for estimating *r*_*max*_ which accounts for juvenile mortality^29^,^30^, unlike previous estimates of *r*_*max*_ for chondrichthyan species^12^,^31^,^32^:


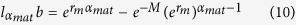


where 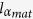 is survival to maturity and is calculated as 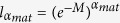, *b* is the annual reproductive output of daughters, *α*_*mat*_ is age at maturity in years, and *M* is the instantaneous natural mortality. We then solve equation 10 for *r*_*max*_ using the nlm.imb function in R. To account for uncertainty in input parameters, we use a Monte Carlo approach and draw values from parameter distributions to obtain 10,000 estimates of *r*_*max*_. Next we describe how we determined each parameter distribution.

#### Annual reproductive output (*b*)

Adult female mobulid rays only have one active ovary and uterus where a single pup grows. This sets the upper bound of annual fecundity to one pup per year^14^, and assuming a 1:1 sex ratio, results in an estimate of *b* of 0.5 female pups per year. It is possible female devil rays have a biennial cycle of reproduction where one pup is produced every two years, as in manta rays^12^, so the lower bound for our estimate of *b* is 0.25 female pups per year. Thus we draw *b* from a uniform distribution bound between 0.25 and 0.5. While it is possible that a very small percentage of litters could consist of two pups, there has not been any cases of two pups being observed in any devil ray, and only a single confirmed case in the Reef Manta Ray (*Manta alfredi*)^33^.

#### Age at maturity (*α*_*mat*_)

There are no direct estimates of age at maturity for any mobulid ray, but using age and growth data from Cuevas-Zimbrón^13^ and a size 50% at maturity of 2000 mm DW from Serrano-López^34^, we assume female *M. japanica* individuals reach knife-edge maturity sometime between 5 and 6 years. Thus we draw *α*_*mat*_ from a uniform distribution bound between 5 and 6.

#### Natural mortality (*M*)

We estimate natural mortality as the reciprocal of average lifespan: *M* = 1/*ω* where average lifespan *ω* is (*α*_*mat*_ + *α*_*max*_)/2). We used this *M* estimate to calculate survival to maturity 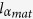 as 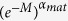. This method produces realistic estimates of *r*_*max*_ when accounting for survival to maturity, unlike many other commonly used natural mortality estimators^29^. We also use our estimate of *Z* from Part 2, which represents both natural and fishing mortality, to contextualize our estimate of *M*. More specifically, our estimate of *M* needs to be lower than our estimate of *Z* to be credible. We calculated maximum age (*α*_*max*_) based on the results of our analysis in Part 1 and estimated it to be between 15 and 20 years. We therefore calculate *M* iteratively by drawing values of *α*_*mat*_ (described in the section above) and *α*_*max*_ from uniform distributions bound between the ranges mentioned.

### Part 4. Comparison of devil ray *r*_*max*_ among chondrichthyans

We re-estimate *r*_*max*_ for the 94 chondrichthyans with complete life history data examined in^32^,^12^,^29^ using Equation 10. We also update estimates of *r*_*max*_ for manta rays (*Manta* spp.)^12^ as a comparison with a closely related species.

## Results

### Part 1: Re-fitting the growth curve for *Mobula japanica*

**Figure 1:**
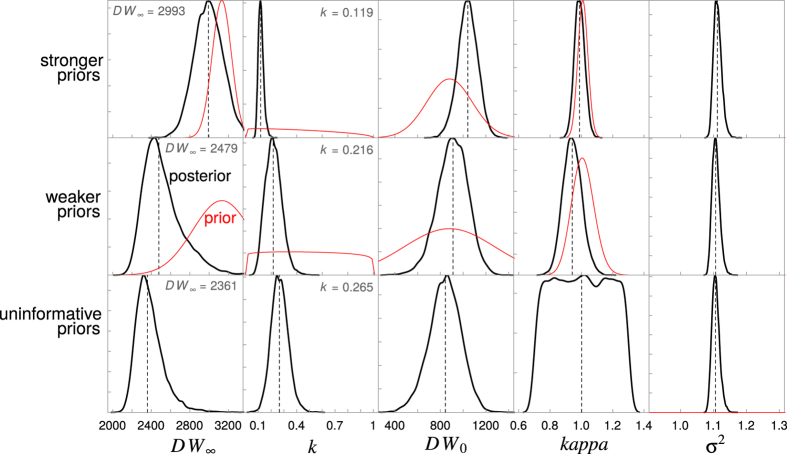
Prior and posterior distributions for the Spinetail Devil Ray (*Mobula japanica*) von Bertalanffy growth parameters (*DW*_∞_, *k* and *DW*_0_) and the error term (σ^2^) for the three Bayesian models with strong, weaker, and uninformative priors. Median values are shown by the dashed lines, posterior distributions by the black lines, and prior distributions by the red lines. No prior distributions are shown when priors are uninformative (uniform distribution).

**Figure 2:**
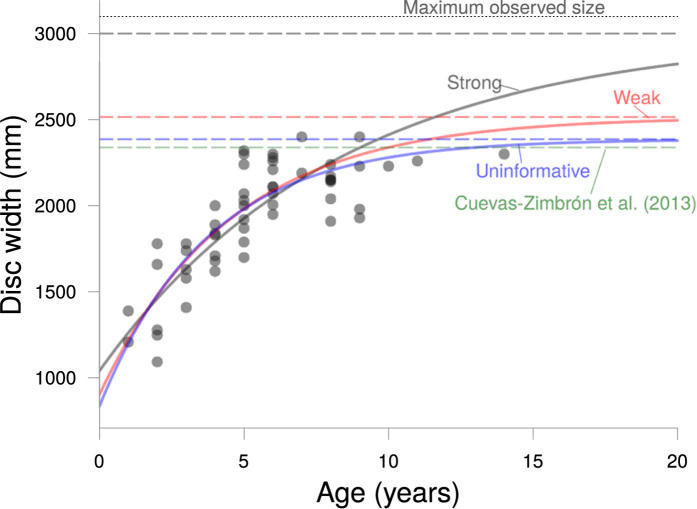
Length-at-age data for the Spinetail Devil Ray (*Mobula japanica*) showing the Bayesian von Bertalanffy growth curve fits for models with strong (grey), weaker (red), and uninformative (blue) priors, as well as the original model fit from Cuevas-Zimbrón *et al*.^13^. Dashed lines show the asymptotic size (*DW*_∞_) estimates for each model. Dotted line represents the maximum known size for the species.

The Bayesian model with strong priors yielded a lower estimate of *k* (0.12 year^−1^) and a higher estimate of *DW*_∞_ (2995 mm DW) than the estimates based on weaker and uninformative priors (Table 2, Fig. 1). The asymptotic size in the model with strong priors was closest to the maximum observed size for this species (Fig. 2). Estimates of *k* were lowest in the model with strong priors and highest in the model with uninformative priors (Table 2, Fig. 1).

### Part 2. Estimating total mortality using the catch curve

**Figure 3:**
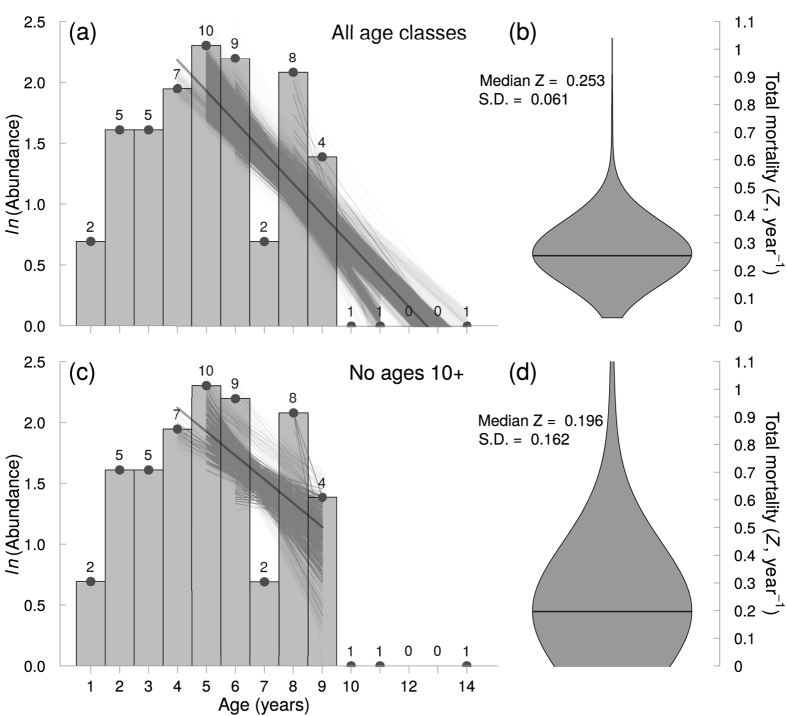
Estimation of total mortality *Z* from bootstrapped catch composition data for the Spinetail Devil Ray (*Mobula japanica*) from Cuevas-Zimbrón *et al.*^13^ using (**a,b**) all data points and (**c,d**) exluding ages 10 and older. (**a,c**) Catch curve of natural log abundance at age. The regression lines represent the estimated slopes when omitting different age-class subsets (as shown by the horizontal extent of each line), and resampling 80% of the data. Note that individual estimates of *Z* differ in the number of age-classes included for its computation, resulting in regression lines of different lengths. (**b,d**) Violin plot of estimated total mortality (*Z*) values calculated using the bootstrap resampling method. Estimates from different age-classes suggest an estimate of *Z* ≈ 0.25 year^−1^. The median is shown by the dark grey line.

Our catch curve analysis, using all data points, yielded a median estimate of *Z* = 0.254 year^−1^, with 95% of bootstrapped estimates ranging between 0.210 and 0.384 year^−1^ (Fig. 3a,b). When removing individuals aged 10 and older, our catch curve anaysis resulted in a median estimate of *Z* = 0.196 year^−1^, albeit with a higher uncertainty than our estimate with all data points (Fig. 3c,d). Assuming *Z* is approximately 0.2 year^−1^ is therefore a relatively conservative estimate of total mortality; we infer natural mortality *M* of *M. japanica* must be less than 0.2 year^−1^.

One remaining question is how our assumption of constant selectivity affects the catch curve in Fig. 3. To explore the effects of differences in *Z* given constant selectivity we simulated a population with the life history of *M. japanica* and show that steep declines of older individuals are possible by fishing alone (see Supplementary material).

By substracting our distribution of natural mortality *M* estimates from our distribution of total mortality *Z* from the catch curve excluding ages 10 and older we obtain a distribution of fishing mortality values, which resulted in a median estimate of *F* = 0.110 year^−1^, albeit with a high degree of uncertainty (95^th^ percentile = −0.034–0.610).

### Part 3. Maximum population growth rate *r*_*max*_ of the Spinetail Devil Ray

From Part 1, we estimated that maximum lifespan was between 15 and 20 years. Combining this with estimated age at maturity, the median estimates of average lifespan for the Spinetail Devil Ray was 11.5 years, and therefore the median natural mortality *M* estimate was 0.087 year^−1^ (95th percentile = 0.079–0.097). Using this information to create a bounded distribution for natural mortality in equation 10, we found the median maximum intrinsic rate of population increase *r*_*max*_ for devil rays is 0.077 year^−1^ (95^th^ percentile = 0.042–0.108).

### Part 4. Comparing *devil ray r*_*max*_ to other chondrichthyans

**Figure 4:**
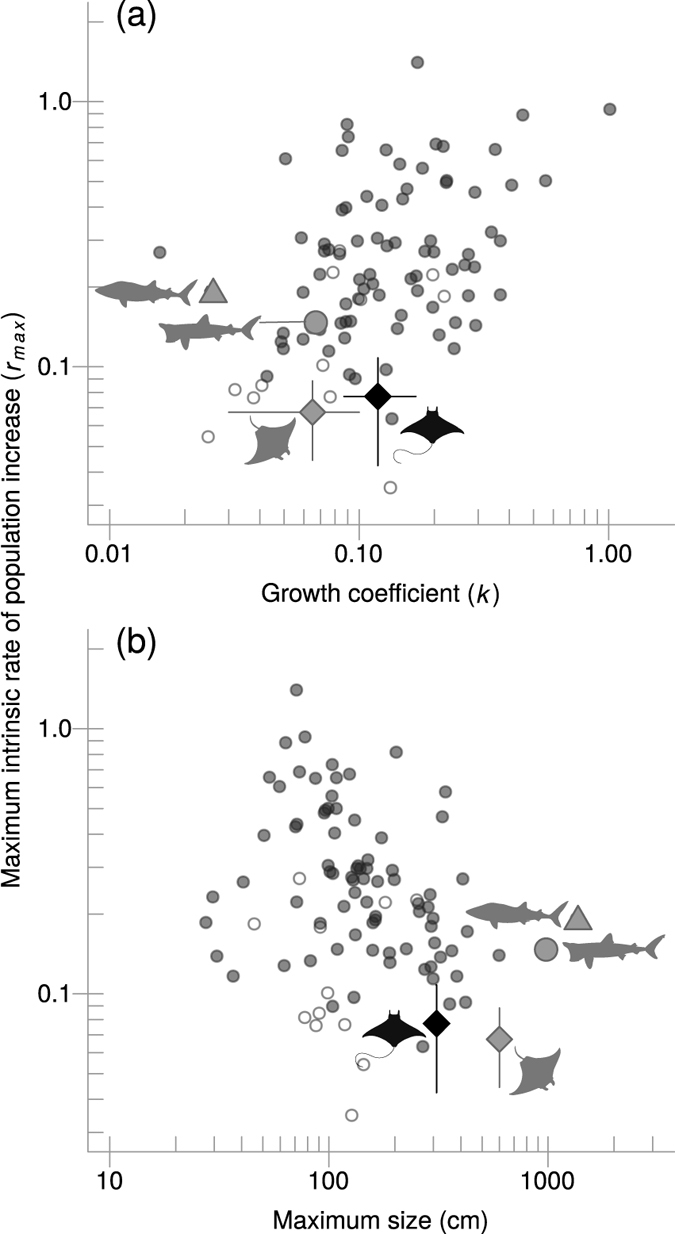
Comparison of maximum intrinsic rate of population increase (*r*_*max*_) for 96 elasmobranch species arranged by (**a**) growth coefficient *k* and (**b**) maximum size. Small open circles represent deep sea species while small grey circles denote oceanic and shelf species. Four species are highlighted using silhouettes and larger symbols: the Spinetail Devil Ray (*Mobula japanica*) is shown by the black diamond, while the manta ray (*Manta* spp.), Whale Shark (*Rhincodon typus*) and Basking Shark (*Cetorhinus maximus*) are represented by the grey diamond, triangle and circle, respectively. Sources and attributions for the silhouettes used are as follows. Devil ray: Public Domain. Available at http://phylopic.org/image/914af720-568d-47ef-ada8-ed5a10660266/. Manta ray: Icon made by http://www.freepik.comFreepik from http://www.flaticon.comFlaticon. Available at http://www.flaticon.com/free-icon/manta-ray-shape_47440. Whale Shark: by Scarlet23 (vectorized by T. Michael Keesey), licensed under CC BY-SA 3.0 (https://creativecommons.org/licenses/by-sa/3.0/us/). Available at http://phylopic.org/image/ef0b81ab-a39e-4780-a572-e2849a23226b/. Basking Shark: by http://alphateck.com/Nick Botner, freely available online at http://alphateck.com/free-vector-file-20-shark-silhouettes/.

Devil and manta rays have low intrinsic rate of population increase relative to other chondrichthyan species (Fig. 4). Among species with similar somatic growth rates, the Spinetail Devil Ray has the lowest *r*_*max*_ value (black diamond in Fig. 4a). This contrast is strongest when excluding deep-water chondrichthyans (white circles in Fig. 4), which tend to have much lower rates of population increase than shallow-water species^35^. Our estimation of *r*_*max*_ for manta rays (grey diamond in Fig. 4) are comparable with our estimates for the Spinetail Devil Ray, albeit slightly lower (median of 0.068 year^−1^, 95th percentile = 0.045–0.088). Values of *r*_*max*_ for other large planktivorous elasmobranchs (Whale and Basking Sharks) are relatively high compared to manta and devil rays.

## Discussion

In this study, we examined multiple lines of evidence that suggest devil rays have relatively low productivity, and hence high risk of extinction compared to other chondrichthyans. The *r*_*max*_ of the Spinetail Devil Ray is comparable to that of manta rays, and much lower than that of other large planktivorous shallow-water chondrichthyans such as the Whale Shark and the Basking Shark (Fig. 4). We conclude the comparable extinction risks of devil and manta rays, coupled with the ongoing demand for their gill plates and the potential for increasing exploitation of devil rays resulting from manta ray regulation, suggest that conferring a similar degree of protection to all mobulids is warranted.

Our approach provides an alternative to previous data-poor methods for estimating *r*_*max*_ that better captures the effects differing reproductive outputs and juvenile survival have on productivity^29^. This is markedly different to other approaches to estimate *r*_*max*_ that do not take into account reproductive output such as the demographic invariant method^36^, or the rebound potential method^37^ which effectively ignores differences in fecundity and thus does not account for reproduction-related productivity differences^38^. Regardless, most of these life history-based approaches perform similarly for slow growing species^30^. When catch trends are available in addition to life history parameters, other approaches can be used (e.g. refs 39 and 40).

The comparable low productivities of devil and manta rays, regardless of differences in body size, are largely due to their very low reproductive rates. Mobulid rays have at most a single pup annually or even biennially, while the Whale Shark can have litter sizes of up to 300 pups^41^, thus increasing the potential ability of this species to replenish its populations (notwithstanding differences in juvenile mortality). The Basking Shark has a litter size of six pups, which partly explains why its *r*_*max*_ is intermediate between mobulids and the Whale Shark. While mobulids mature relatively later with respect to their total lifespan than Basking Sharks and relatively earlier than Whale Sharks, they have lower lifetime fecundity than both Whale and Basking Sharks, limiting their productivity.

**Figure 5:**
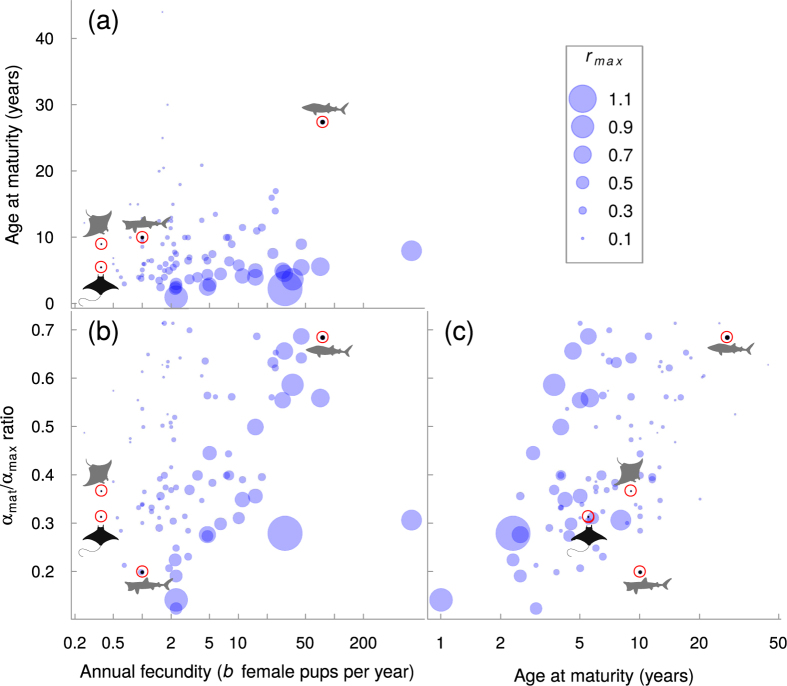
Annual fecundity (*b*, in log-scale) vs (**a**) age at maturity and (**b**) the *α*_*mat*_/*α*_*max*_ ratio. (**c**) Age at maturity vs *α*_*mat*_/*α*_*max*_ ratio. Circle size represent *r*_*max*_ estimates. Four species are highlighted by the red circles and silhouettes: the Spinetail Devil Ray (*Mobula japanica*) is shown by the black silhouette, while the manta ray (*Manta* spp.), Whale Shark (*Rhincodon typus*) and Basking Shark (*Cetorhinus maximus*) are represented by the grey silhouettes. Sources and attributions for the silhouettes used are as follows. Devil ray: Public Domain. Available at http://phylopic.org/image/914af720-568d-47ef-ada8-ed5a10660266/. Manta ray: Icon made by http://www.freepik.comFreepik from http://www.flaticon.comFlaticon. Available at http://www.flaticon.com/free-icon/manta-ray-shape_47440. Whale Shark: by Scarlet23 (vectorized by T. Michael Keesey), licensed under CC BY-SA 3.0 (https://creativecommons.org/licenses/by-sa/3.0/us/). Available at http://phylopic.org/image/ef0b81ab-a39e-4780-a572-e2849a23226b/. Basking Shark: by http://alphateck.com/Nick Botner, freely available online at http://alphateck.com/free-vector-file-20-shark-silhouettes/.

Our results are consistent with the correlation between low somatic growth rates, later maturation, large sizes, and elevated extinction risk^42^,^43^ (Figs 4a and 5). Similar relationships have been found in other marine fishes. For example, in tunas and their relatives, somatic growth rate is the best predictor of overfishing, such that species with slower growth are more likely to be overfished as fishing mortality increases than species with faster growth^44^, likely because the species that grow faster mature earlier. Among the chondrichthyans we compared, species with low fecundities have an elevated extinction risk regardless of their age at maturity (Fig. 5a).

Our method for estimating growth rate for the Spinetail Devil Ray provided lower estimates of the growth coefficient *k* than was reported in the original study^13^, especially when we used strongly informative priors. When using strong priors the estimated asymptotic size was very close to the expectation of it being 90% of maximum size. On the other hand, our scenario with uninformative priors provided growth coefficient *k* estimates that are very similar to the original estimates, which were obtained by nonlinear least squares minimization (Fig. 2b). Our growth estimates from the model with strong priors are consistent with our expected values of *k* if length-at-age data were available for larger individuals. Given that the length-at-age data available only includes individuals up to two-thirds of the maximum size recorded for *M. japanica*, we believe that our approach provides more plausible estimates of growth rates when data are sparse. Our approach provides further evidence that Bayesian estimation is useful for data-sparse species as the available life history information can be easily incorporated in the form of prior distributions, particularly when missing samples of the largest or smallest individuals^15^. Incorporating prior information when fitting growth curves is an alternative to fixing model parameters, which often biases growth estimates^45^. In other words, using Bayesian inference allows us to incorporate out-of-sample knowledge of observed maximum sizes and sizes at birth, thus improving our estimates of growth rates and asymptotic size^15^.

The lower estimate of fishing mortality we calculated from the catch curve (*Z* − *M* = *F* = 0.110 year^−1^) is higher than our estimate of *r*_*max*_, which also represents the fishing mortality *F* expected to drive this species to extinction (*F*_*ext*_ = 0.077 year^−1^)^28^. Even though our estimates of total and fishing mortality are highly uncertain (Fig. 3b,d), our estimate of *Z* was derived excluding age classes that might have been underrepresented, resulting in conservative estimates of both total and fishing mortalities. Hence we infer that before the fishery ceased in 2007, the Spinetail Devil Ray population we examined was probably being fished unsustainably at a rate high enough to lead to eventual depletion. Many teleost fisheries support fishing mortalities that are many times larger than natural mortality. However, mobulids likely have low capacity to compensate for fishing, because their large offspring and low fecundity suggest weak density-dependent regulation of populations^46^,^47^.

The steep decline in abundance of individuals older than age 9 is a pattern that could emerge solely from fishing (see Supplementary material), migration of larger individuals away from the fishing grounds, or as a result of differences in catchability between age groups. To reduce the likelihood of overestimating *Z* because of differences in catchability or migration, we estimated a slope of the catch curve excluding these age groups. This more conservative estimate of *Z*, albeit being more uncertain, better represents the total mortality of the population by discarding the age classes that could bias our estimate of *Z*.

The major caveats of using a catch curve analysis to estimate total mortality are that it assumes there is no size selectivity in catch, recruitment is constant, the population is closed, and that the catch is a large enough sample to sufficiently represent population age structure. These assumptions are also required in age and growth studies when using length-at-age data. Thus, the use of catch curves to estimate fishing mortality could be applied to other chondrichthyan growth studies, assuming that sampling is not systematically size selective and that metapopulation dynamics are not influencing the sample. Whether or not this latter assumption is valid for highly migratory elasmobranch species has yet to be tested.

Unregulated small-scale artisanal fisheries are targeting mobulids throughout the world^4^,^5^. Our findings strongly indicate that there is little scope for unmanaged artisanal fisheries to support sustainable international exports of gill plates or even domestic meat markets. Furthermore, the unsustainable fishing mortality stemming from the removal of relatively few individuals by an artisanal fishery suggests we urgently need to understand the consequences of bycatch of mobulid rays in industrial trawl, long line and purse seine fisheries^4^,^8^. The combination of high catch rates and low post-release survival suggest fishing mortality rates need to be understood and potentially minimized to ensure the future persistence of these species.

## Additional Information

**How to cite this article**: Pardo, S. A. *et al.* Growth, productivity, and relative extinction risk of a data-sparse devil ray. *Sci. Rep.* **6**, 33745; doi: 10.1038/srep33745 (2016).

## Supplementary Material

Supplementary Information

## Figures and Tables

**Table 1:** Priors used in the three different Bayesian von Bertalanffy growth models.

Parameter	Strong priors	Weaker priors	Uninformative priors
*k*	*beta* (1.05, 1.5)	*Beta* (1.05, 1.1)	*Uniform* (0, 2)
*DW*_∞_	*normal* (3100 * *kappa*, 100)	*Normal* (3100 * *kappa*, 400)	*Uniform* (0, 4000)
*DW*0	*normal* (880, 200)	*Normal* (880, 300)	*Uniform* (0, 2000)
*kappa*	*gamma* (1000, 990)	*Gamma* (200, 198)	*Uniform* (0.7, 1.3)
σ^2^	*half cauchy* (0, 30000)	*halfCauchy* (0, 30000)	*halfCauchy* (0, 30000)

**Table 2:** Mean von Bertalanffy growth parameter estimates for the three Bayesian models with differing priors.

Model	Estimate of *DW*_∞_	Estimate of *k*	Estimate of *σ*^2^
Strong priors	2999 mm [2711–3295]	0.12 year^−1^ [0.086-0.169]	0.106 [0.088–0.13]
Weaker priors	2515 mm [2232–3018]	0.221 year^−1^ [0.11-0.353]	0.102 [0.084–0.124]
Uninformative priors	2386 mm [2175–2744]	0.268 year^−1^ [0.144-0.406]	0.102 [0.084–0.124]

Values inside square brackets are the 95% credible intervals (CI).

## References

[b1] O’Malley M. P., Lee-Brooks K. & Medd H. B. The global economic impact of manta ray watching tourism. PLoS ONE 8, e65051 (2013). 23741450 10.1371/journal.pone.0065051 PMC3669133

[b2] Mundy-Taylor V. & Crook V. Into the deep: Implementing CITES measures for commercially-valuable sharks and manta rays. Tech. Rep. TRAFFIC, Cambridge (2013).

[b3] Zeeberg J., Corten A. & de Graaf E. Bycatch and release of pelagic megafauna in industrial trawler fisheries off Northwest Africa. Fisheries Research 78, 186–195 (2006).

[b4] Croll D. A. *et al.* Vulnerabilities and fisheries impacts: the uncertain future of manta and devil rays. Aquatic Conservation: Marine and Freshwater Ecosystems 26, 562–575 (2016).

[b5] Couturier L. I. E. *et al.* Biology, ecology and conservation of the Mobulidae. Journal of Fish Biology 80, 1075–1119 (2012). 22497374 10.1111/j.1095-8649.2012.03264.x

[b6] Ejecutivo Poder Federal. NOM-029-PESC-2006, Responsible Fisheries of Sharks and Rays, Specifications for their Use (in Spanish) (2007).

[b7] Heinrichs S., O’Malley M., Medd H. & Hilton P. Manta Ray of Hope 2011 Report: The Global Threat to Manta and Mobula Rays. Tech. Rep. WildAid, San Francisco, CA (2011).

[b8] Francis M. P. & Jones E. G. Movement, depth distribution and survival of spinetail devilrays (*Mobula japanica*) tagged and released from purse-seine catches in New Zealand. Aquatic Conservation: Marine and Freshwater Ecosystems available online (2016).

[b9] Musick J. A. Criteria to Define Extinction Risk in Marine Fishes: The American Fisheries Society Initiative. Fisheries 24, 6–14 (1999).

[b10] Reynolds J. D., Dulvy N. K., Goodwin N. B. & Hutchings J. A. Biology of extinction risk in marine fishes. Proceedings of the Royal Society B 272, 2337–44 (2005). 16243696 10.1098/rspb.2005.3281 PMC1559959

[b11] Jennings S., Reynolds J. D. & Mills S. C. Life history correlates of responses to fisheries exploitation. Proceedings of the Royal Society B 265, 333–339 (1998).

[b12] Dulvy N. K., Pardo S. A., Simpfendorfer C. A. & Carlson J. K. Diagnosing the dangerous demography of manta rays using life history theory. PeerJ 2, e400 (2014). 24918029 10.7717/peerj.400 PMC4045333

[b13] Cuevas-Zimbrón E., Sosa-Nishizaki O., Pérez-Jiménez J. & O’Sullivan J. An analysis of the feasibility of using caudal vertebrae for ageing the spinetail devilray, Mobula japanica (Müller and Henle, 1841). Environmental Biology of Fishes 96, 907–914 (2013).

[b14] Notarbartolo-Di-Sciara G. A revisionary study of the genus *Mobula* Rafinesque, 1810 (Chondrichthyes: Mobulidae) with the description of a new species. Zoological Journal of the Linnean Society 91, 1–91 (1987).

[b15] Siegfried K. & Sansó B. Two Bayesian methods for estimating parameters of the von Bertalanffy growth equation. Environmental Biology of Fishes 77, 301–308 (2006).

[b16] White W. T., Giles J. & Potter I. C. Data on the bycatch fishery and reproductive biology of mobulid rays (Myliobatiformes) in Indonesia. Fisheries Research 82, 65–73 (2006).

[b17] White W. T. *et al.* Economically important sharks and rays of Indonesia (Australian Centre for International Agricultural Research, Canberra, 2006).

[b18] Paulin C. D., Habib G., Carey C. L., Swanson P. M. & Voss G. J. New records of *Mobula japanica* and *Masturus lanceolatus*, and further records of *Luvaris imperialis* (Pisces: Mobulidae, Molidae, Louvaridae) from New Zealand. New Zealand Journal of Marine and Freshwater Research 16, 11–17 (1982).

[b19] Poortvliet M. *et al.* A dated molecular phylogeny of manta and devil rays (Mobulidae) based on mitogenome and nuclear sequences. Molecular Phylogenetics and Evolution 83, 72–85 (2015). 25462995 10.1016/j.ympev.2014.10.012

[b20] Froese R. & Binohlan C. Empirical relationships to estimate asymptotic length, length at first maturity and length at maximum yield per recruit in fishes, with a simple method to evaluate length frequency data. Journal of Fish Biology 56, 758–773 (2000).

[b21] Stan Development Team. Stan Modeling Language User’s Guide and Reference Manual, Version 2.7.0, URL http://mc-stan.org/ ( 2015).

[b22] Stan Development Team. Stan: A C++ Library for Probability and Sampling, Version 2.7.0, URL http://mc-stan.org/ ( 2015).

[b23] R Core Team. R: A Language and Environment for Statistical Computing, URL https://www.r-project.org/ (2015).

[b24] Thorson J. T. & Prager M. H. Better catch curves: Incorporating age-specific natural mortality and logistic selectivity. Transactions of the American Fisheries Society 140, 356–366 (2011).

[b25] Hordyk A., Ono K., Valencia S., Loneragan N. & Prince J. A novel length-based empirical estimation method of spawning potential ratio (SPR), and tests of its performance, for small-scale, data-poor fisheries. ICES Journal of Marine Science: Journal du Conseil 72, 217–231 (2015).

[b26] Ricker W. Computation and interpretation of biological statistics of fish populations. Tech. Rep. Department of the Environment Fisheries and Marine Service, Ottawa (1975).

[b27] Charnov E. L. & Schaffer W. M. Life-History Consequences of Natural Selection: Cole’s Result Revisited. The American Naturalist 107, 791–793 (1973).

[b28] Myers R. A. & Mertz G. The limits of exploitation: A precautionary approach. Ecological Applications 8, 165–169 (1998).

[b29] Pardo S. A., Kindsvater H. K., Reynolds J. D. & Dulvy N. K. Maximum intrinsic rate of population increase in sharks, rays, and chimaeras: the importance of survival to maturity. Canadian Journal of Fisheries and Aquatic Sciences 73, 1159–1163 (2016).

[b30] Cortés E. Perspectives on the intrinsic rate of population growth. Methods in Ecology and Evolution, available online (2016).

[b31] Hutchings J. A., Myers R. A., García V. B., Lucifora L. O. & Kuparinen A. Life-history correlates of extinction risk and recovery potential. Ecological Applications 22, 1061–1067 (2012). 22827118 10.1890/11-1313.1

[b32] García V. B., Lucifora L. O. & Myers R. A. The importance of habitat and life history to extinction risk in sharks, skates, rays and chimaeras. Proceedings of the Royal Society B 275, 83–89 (2008). 17956843 10.1098/rspb.2007.1295 PMC2562409

[b33] Marshall A. D. & Bennett M. B. Reproductive ecology of the reef manta ray *Manta alfredi* in southern Mozambique. Journal of Fish Biology 77, 169–190 (2010). 20646146 10.1111/j.1095-8649.2010.02669.x

[b34] Serrano-López J. *Estudio comparativo de la reproducción de tres especies del género* Mobula *(Chondrichthyes: Mobulidae) en el suroeste del Golfo de California, México.* MSc thesis, BCS, La Paz (2009).

[b35] Simpfendorfer C. A. & Kyne P. M. Limited potential to recover from overfishing raises concerns for deep-sea sharks, rays and chimaeras. Environmental Conservation 36, 97–103 (2009).

[b36] Niel C. & Lebreton J.-D. Using demographic invariants to detect overharvested bird populations from incomplete data. Conservation Biology 19, 826–835 (2005).

[b37] Au D. W. & Smith S. E. A demographic method with population density compensation for estimating productivity and yield per recruit of the leopard shark (*Triakis semifasciata*). Canadian Journal of Fisheries and Aquatic Sciences 54, 415–420 (1997).

[b38] Au D. W., Smith S. E. & Show C. New abbreviated calculation for measuring intrinsic rebound potential in exploited fish populations–example for sharks. Canadian Journal of Fisheries and Aquatic Sciences 72, 767–773 (2015).

[b39] Dick E. J. & MacCall A. D. Depletion-Based Stock Reduction Analysis: A catch-based method for determining sustainable yields for data-poor fish stocks. Fisheries Research 110, 331–341 (2011).

[b40] Carruthers T. R. *et al.* Evaluating methods for setting catch limits in data-limited fisheries. Fisheries Research 153, 48–68 (2014).

[b41] Joung S.-J., Chen C.-T., Clark E., Uchida S. & Huang W. Y. P. The whale shark, *Rhincodon typus*, is a livebearer: 300 embryos found in one ‘megamamma’ supreme. Environmental Biology of Fishes 46, 219–223 (1996).

[b42] Jennings S. & Dulvy N. K. Beverton and Holt’s Insights into Life History Theory: Influence, Application and Future Use. In Payne A. I., Cotter A. J. R. & Potter E. C. E. (eds) Advances in Fisheries Science, 434–450 (Blackwell Publishing Ltd., Oxford, 2008).

[b43] Dulvy N. K., Sadovy Y. & Reynolds J. D. Extinction vulnerability in marine populations. Fish and Fisheries 4, 25–64 (2003).

[b44] Juan-Jordá M. J., Mosqueira I., Freire J. & Dulvy N. K. Life history correlates of marine fisheries vulnerability: a review and a test with tunas and mackerel species. In Briand F. (ed.) Marine extinctions - patterns and processes. CIESM Workshop Monograph no. 45, 113–128 (CIESM Publisher, Monaco, 2013).

[b45] Pardo S. A., Cooper A. B. & Dulvy N. K. Avoiding fishy growth curves. Methods in Ecology and Evolution 4, 353–360 (2013).

[b46] Forrest R. E. & Walters C. J. Estimating thresholds to optimal harvest rate for long-lived, low-fecundity sharks accounting for selectivity and density dependence in recruitment. Canadian Journal of Fisheries and Aquatic Sciences 66, 2062–2080 (2009).

[b47] Kindsvater H. K., Mangel M., Reynolds J. D. & Dulvy N. K. Ten principles from evolutionary ecology essential for effective marine conservation. Ecology and Evolution 6, 2125–2138 (2016). 27069573 10.1002/ece3.2012 PMC4782246

