# Supplementary material for: Growth, productivity, and relative extinction risk of a data-sparse devil ray
Source: Sci Rep. 2016 Sep 23;6:33745. doi: 10.1038/srep33745 (PMC5034314; doi:10.1038/srep33745)
Supplement: Supplementary Information [file srep33745-s1.pdf]

Supplementary materials

for

Growth, productivity, and relative extinction risk of a data-sparse devil ray

Sebastián A. Pardo[1,*], Holly K. Kindsvater[1,2], Elizabeth Cuevas-Zimbrón[3], Oscar Sosa-Nishizaki[4], Juan Carlos Pérez-Jiménez[5], and Nicholas K. Dulvy[1]

[1] Earth to Ocean Research Group, Department of Biological Sciences, Simon Fraser University, Burnaby, BC, Canada

[2] Department of Ecology, Evolution, and Natural Resources, Rutgers University, New Brunswick, NJ, USA

[3] Fisheries Biology Consultant, Boulevard Chahue #66 Mz 6, Sector R, La Crucecita, Bahías de Huatulco, Oaxaca, Mexico

[4] Laboratorio de Ecología Pesquera, Departamento de Oceanografía Biológica, CICESE, Ensenada, Baja California, Mexico

[5] Ciencias de la Sustentabilidad, El Colegio de la Frontera Sur, Lerma, Campeche, Mexico

*Corresponding author. Email: spardo@sfu.ca

Age-structured population modelling

The catch curve analysis makes the assumption that selectivity is constant for rays older than age 3. While it is possible that selectivity is biased by size in this fishery, we wanted to explore how constant selectivity affected the population age structure given the life history of *M. japanica* and our rage of fishing mortality estimates.

To show the relationship between fishing mortality, fishery selectivity, and population age structure, we simulated the deterministic dynamics of an age-structured population of female *M. japanica* with overlapping generations as functions of both age *a* and time *t*. We adapted the age-structured modelling approach in Kindsvater et al. (2016). Model equations and parameter descriptions are given in Table S1; parameter values are given in Table S2. We assumed that age *a* and disc width (which we refer to as *L*) are related according to the von Bertalanffy growth function (see Part 1 results, main text). We can calculate mass-at-age $W(a)$ as a power function of *L*; this allows us track both age structure and biomass dynamics of our simulated population.

Our model follows the standard conventions of age-structured fisheries models (Mangel 2006). Annual egg production of the population $E(t)$ depends on the spawning stock biomass, which we calculate as number of individuals of age a, the probability they are mature at that age $p_m(a)$, and the annual birth rate of female Spinetail Devil Rays f. The age at which 50% of individuals are mature is defined as a_{mat}. We assumed the population dynamics are regulated by density-dependent recruitment, where the number of recruits N_0 is determined by the population growth rate from low densities α and the asymptotic population size, which depends on the parameters α and β in the Beverton-Holt stock-recruitment function (see Supplement 2 of Kindsvater et al. 2016). Lacking data on the stock recruitment relationships of mobulid ray populations, we assumed that the slope of the stock-recruitment function near the origin α (i.e., steepness; Mangel et al. 2010) was less than one, but was large enough for our population to be viable, which we determined through sensitivity analyses. We assumed all recruits are the same size, L_R (in cm), and live to at most A_{max} years. Natural mortality is consistent with our assumptions in the main text, though the estimate is applicable to a population growing without density-dependent mortality, and is likely an underestimate here.

Table S1. Equations used to model the different biological processes in the age-structured population model.

Biological process	Equation	Parameter interpretations
Von Bertalanffy growth function	$L(a+1) = L(a)e^{-k} + L_{\infty}(1 - e^{-k})$.	L_{∞} and k determine size-at-age L
Maturation probability ogive	$p_m(a) = \frac{1}{1+e^{-q(a-a_{mat})}}$	a_{mat} is the age at 50% maturity; ogive steepness set by *q*
Body mass (depends on $L(a)$)	$W(a) = \nu L(a)^{\omega}$	ν and ω are scale and shape parameters
Natural mortality	M	Assumed to be constant
Fishing mortality	$F(a) = \begin{cases} 0 & if\ a < 3 \\ 1 & if\ a \geq 3 \end{cases}$	constant selectivity
Egg production of the population (all mature individuals)	$E(t) = \sum_a N(a,t)\ p_m(a)\, f$	Assumed mobulids produce 1 pup/year, half of which are female
Density dependent recruitment	$N_0(t+1) = \frac{\alpha E(t)}{1+\beta E(t)}$.	Beverton-Holt recruitment function
Population dynamics through time	$N(a+1, t+1) = \begin{cases} N_0(t) & if\ a = 0 \\ N(a,t)\ e^{-(M(a)+F(a))} & if\ a > 0 \end{cases}$	*N(a,t)* is the number of individuals of age *a* alive at time *t*

Table S2. Life history parameters used for *M. japanica*.

A_{max}	a_{mat}	L_{∞} (cm)	k	f	M	α	β
18	5	299.5	0.3	0.5	0.087	0.9	4.5×10^{-5}

Fig. S1 shows the graphs for the life history functions used to simulate the dynamics of the population through time t.

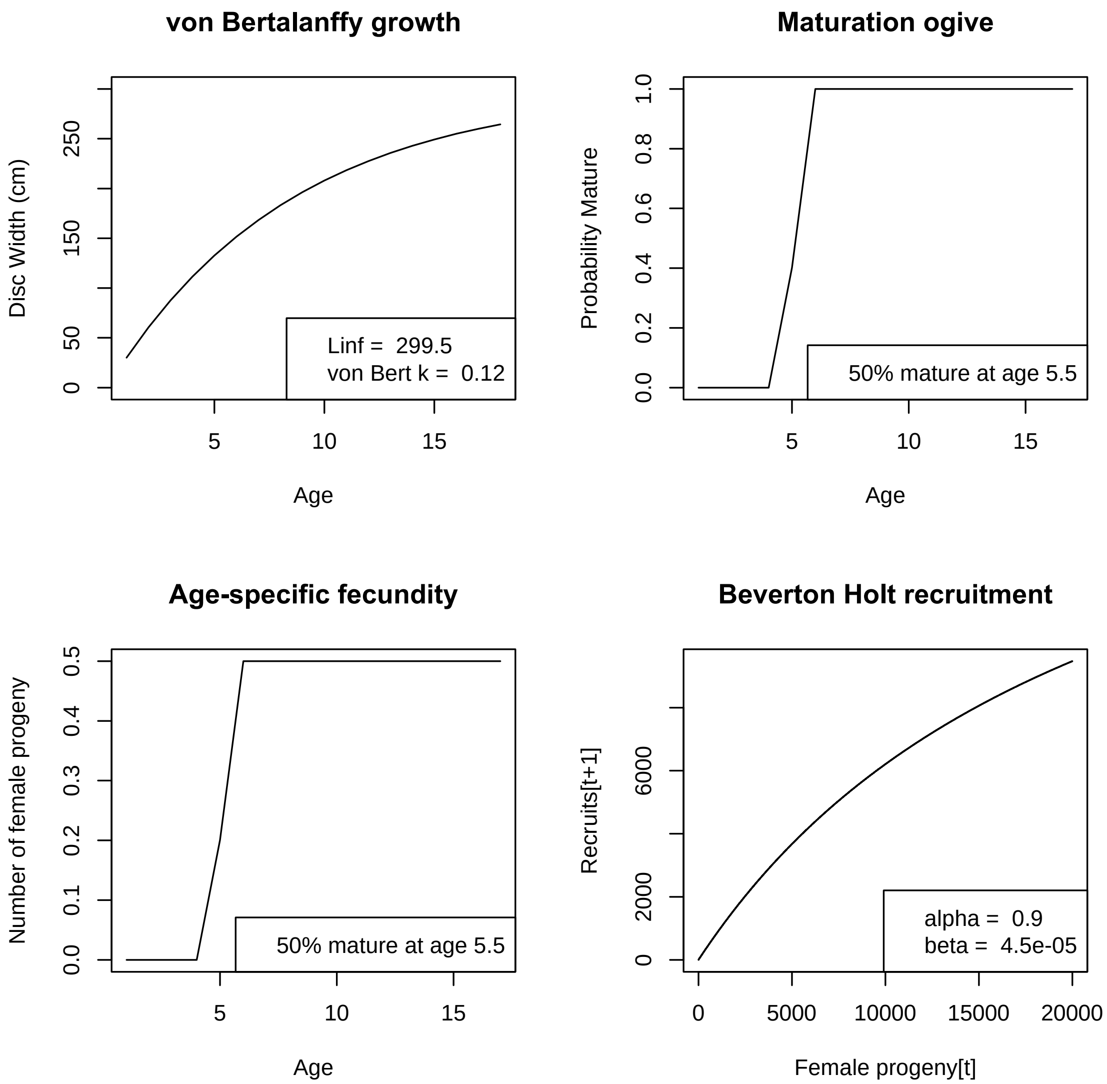

Figure S1. Life history functions that define growth, maturation, fecundity and recruitment for *M. japanica* (see Table S1 and Table S2). These functions are used in the age-structured population dynamics model used to generate Figs. S2-S3.

We simulated the population for several generations in the absence of fishing until it reached a stable age distribution (equilibrium). The proportion of individuals in each age class is constant at this point. We then added fishing mortality, again assuming constant selectivity for ages 3 and above. We varied fishing mortality F according to the range of estimates we found with our catch curve analysis (Part 2, main text).

In Fig. S2 we show the stable age distribution with and without fishing for the lower value of our estimate of fishing mortality F. In Fig. S3, we show the same distribution with the upper value of our estimate of fishing mortality F. Notice that with constant selectivity for disc widths greater than 800 mm or 2 years of age, we are able to recreate the demographic pattern in our catch curve (Part 2, main text). This is because the population quickly moves into a stable age distribution; fishing the younger age classes thus erodes the number of individuals in larger age classes.

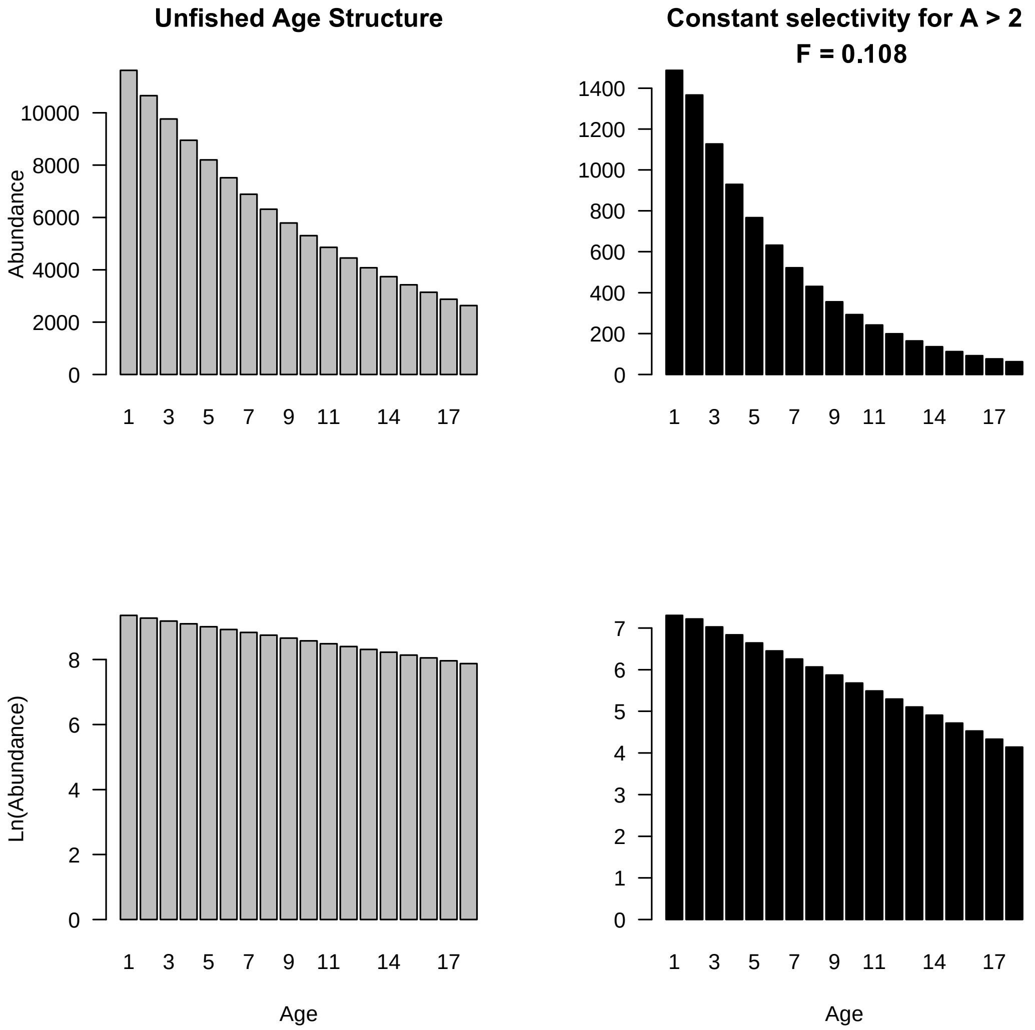

Figure S2. The stable age distribution before and after fishing. Both raw abundance (top row) and the natural log of abundance (bottom row) are plotted. Fishery selectivity was assumed to be constant after age 3, when *M. japanica* disc widths are greater than 80 cm (upper left panel, Fig. S1). In this case, fishing mortality *F* was 0.108, which is the lower limit on our estimate of *F* from the catch curve (main text).

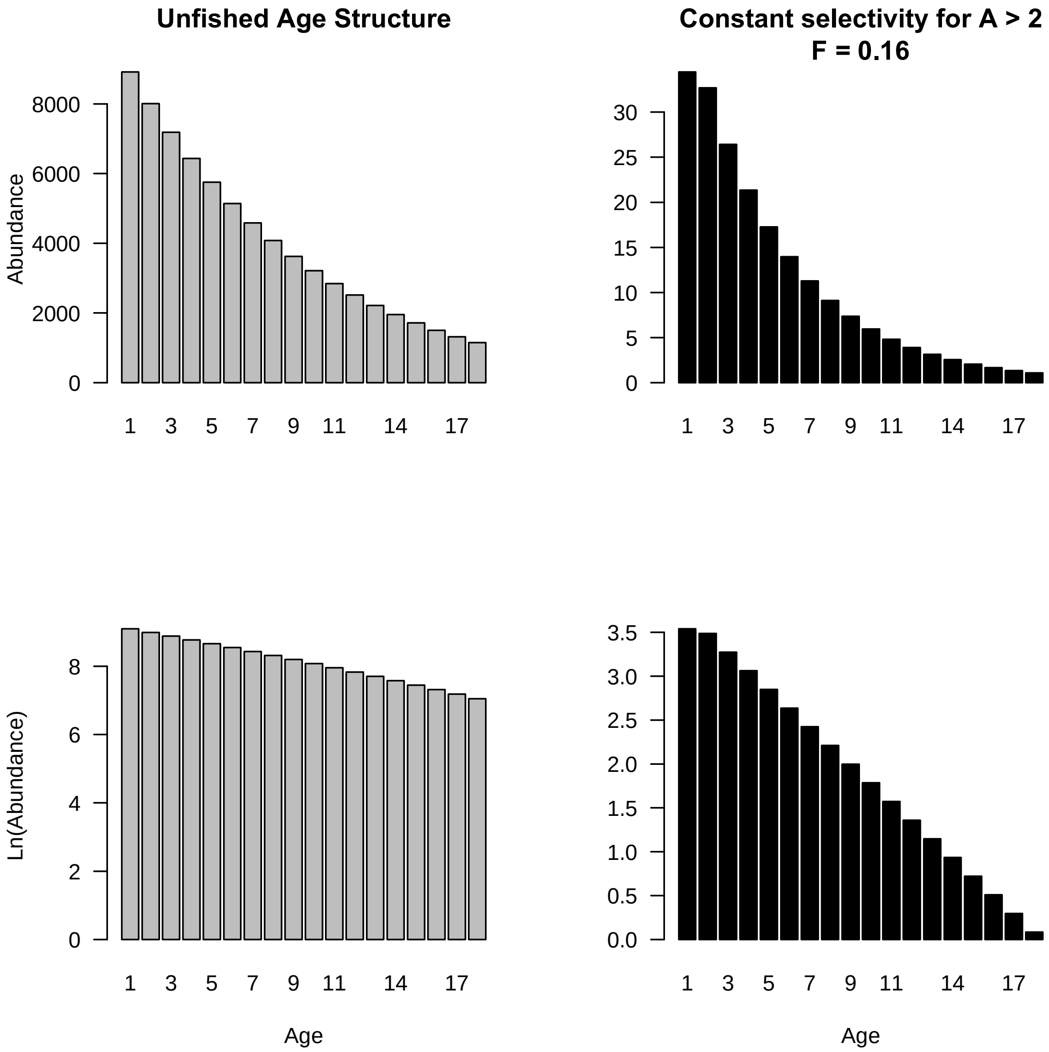

Figure S3. The age distribution before and after fishing. This model run was identical to Figure S2, except fishing mortality was 0.16, which is the upper limit on our estimate of *F* from the catch curve (main text). This run of the model generates the age distribution most similar to that of the observed catch curve (Part 2, main text).

References

Kindsvater, H. K.**,** Mangel, M., Reynolds, J. D. & Dulvy, N.K. Ten principles from evolutionary ecology essential for effective marine conservation*. Ecology and Evolution* **6,** 2125–2138 (2016).

Mangel, M. The Theoretical Biologist's Toolbox*.* Cambridge University Press, New York, NY, USA (2006).

Mangel, M., Brodziak, J. K. T. & DiNardo, G. Reproductive ecology and scientific inference of steepness: a fundamental metric of population dynamics and strategic fisheries management. *Fish and Fisheries* **11**, 89-104 (2010).
